# A new genus and two new species of oonopid spiders from Myanmar (Araneae, Oonopidae)

**DOI:** 10.3897/zookeys.931.49638

**Published:** 2020-04-30

**Authors:** Yanfeng Tong, Shuqiang Li

**Affiliations:** 1 Life Science College, Shenyang Normal University, Shenyang 110034, China Shenyang Normal University Shenyang China; 2 Southeast Asia Biological Diversity Research Institute, Chinese Academy of Sciences, Yezin, Nay Pyi Taw 05282, Myanmar Southeast Asia Biological Diversity Research Institute, Chinese Academy of Sciences Yezin China; 3 Institute of Zoology, Chinese Academy of Sciences, Beijing 100101, China Institute of Zoology, Chinese Academy of Sciences Beijing China

**Keywords:** Goblin spiders, new genus, new species, Southeast Asia, taxonomy

## Abstract

A new genus, *Promolotra***gen. nov.**, including two new species, *P. hponkanrazi***sp. nov.** (♂♀) and *P. shankhaung***sp. nov.** (♂), is described from Myanmar. The new genus is similar to *Molotra* Ubick & Griswold, 2011 but can be distinguished by the completely fused bulb and cymbium, the presence of a receptacle, the absence of grooves connecting either the anterior or posterior pairs of spiracles, and the incised labium of both sexes.

## Introduction

Oonopidae is a diverse spider family with 1846 extant described species in 113 genera (Li 2020). They have a nearly worldwide distribution, occurring mainly in the leaf litter, under bark, and in the tree canopy (Jocqué and Dippenaar-Schoeman 2006; Ubick and Dupérré 2017). Several new oonopid genera of Southeast Asia have been erected in recent years. For example, *Aposphragisma* Thoma, 2014 from Indonesia, Malaysia, Singapore and Vietnam, *Prethopalpus*Baehr et al., 2012 from Indonesia, Malaysia and Singapore, *Sicariomorpha* Ott & Harvey, 2015 from Malaysia, and *Vientianea* Tong & Li, 2013 from Laos (Baehr et al. 2012; Tong and Li 2013; Thoma et al. 2014; Ott et al. 2015). The oonopid fauna of Myanmar has been poorly studied. Up to now, eight species have been reported from Myanmar, i.e., *Gamasomorpha inclusa* (Thorell, 1887), *G. psyllodes* Thorell, 1897, *G. sculptilis* Thorell, 1897, *Kachinia mahmolae* Tong & Li, 2018, *K. putao* Tong & Li, 2018, *Opopaea kanpetlet* Tong & Li, 2020, *O. zhigangi* Tong & Li, 2020, and *Xestaspis parmata* Thorell, 1897 (Tong et al. 2018, 2020; WSC 2020). In this paper, a new oonopid genus and two new species collected from Myanmar, are described and illustrated.

## Materials and methods

The specimens were examined using a Leica M205C stereomicroscope. Details were studied under an Olympus BX51 compound microscope. Photos were made with a Canon EOS 550D zoom digital camera (18 megapixels) mounted on an Olympus BX51 compound microscope. Vulvae were cleared in lactic acid. Scanning electron microscope images (SEM) were taken under high vacuum with a Hitachi TM3030 after critical point drying and gold-palladium coating. All measurements were taken using an Olympus BX51 compound microscope and are in millimeters. The type material is deposited in the Institute of Zoology, Chinese Academy of Sciences in Beijing (IZCAS).

The following abbreviations are used in the text and figures: ALE = anterior lateral eyes; ap = apodeme; bls = brush-like structures; dl = dorsal lobe; hss = horseshoe-shaped sclerite; ldi = labium deep incision; pl = posterior lobe; PLE = posterior lateral eyes; PME = posterior median eyes; pr = posterior receptaculum; tls = tube-like structure; tsc = T-shaped sclerite; vl = ventral lobe.

## Taxonomy

### Promolotra gen. nov.

Taxon classificationAnimaliaAraneaeOonopidae

E8737CFF-5CFF-5DEE-AFD6-8CC566798048

http://zoobank.org/66FBE56C-1887-4984-8DB7-5D50FEAA5371

#### Type species.

*Promolotra shankhaung* sp. nov.

#### Etymology.

The generic name refers to the similarities of this genus and *Molotra* and is feminine in gender.

#### Diagnosis.

*Promolotra* gen. nov. resembles *Molotra* Ubick & Griswold, 2011 (Ubick and Griswold 2011) by the heavily sclerotized dorsal and ventral abdominal scuta, the long spines on legs I and II, and the embolar region, but can be distinguished by the completely fused bulb and cymbium, the presence of a receptacle, the absence of grooves connecting either the anterior or posterior pairs of spiracles, and the incised labium of both sexes. The new genus is also similar to *Costarina* Platnick & Dupérré, 2011 by the heavily sclerotized dorsal and ventral abdominal scuta, the long spines on legs I and II, the absence of grooves connecting either the anterior or posterior pairs of spiracles, and the fused cymbium and bulb, but can be distinguished by the absence of 3 transverse ridges on the sternum, the embolar region which barely extends beyond the tip of the cymbiobulbus, and the incised labium of both sexes. The genus *Costarina* has 3 transverse ridges on the sternum, the embolar region is divided into two black prongs that distinctly extend beyond the tip of the cymbiobulbus, and the labium is not indented at the anterior margin (Platnick and Dupérré 2012).

#### Description.

**Figure 1.**
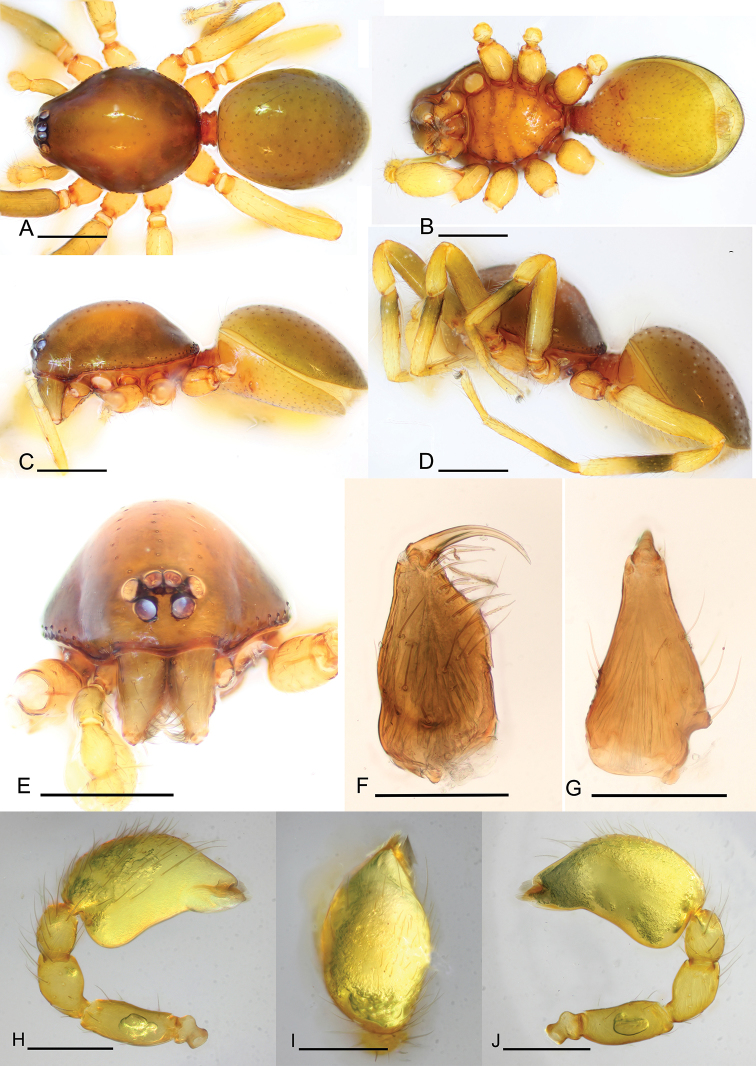
*Promolotra shankhaung* sp. nov., male **A–D** habitus, dorsal, ventral, and lateral views (**D** shows the leg color pattern) **E** prosoma, anterior view **F, G** left chelicera, anterior and lateral views **H–J** left palp, prolateral, dorsal, and retrolateral views. Scale bars: 0.4 mm (**A–E**); 0.2 mm (**F–J**).

**Figure 2.**
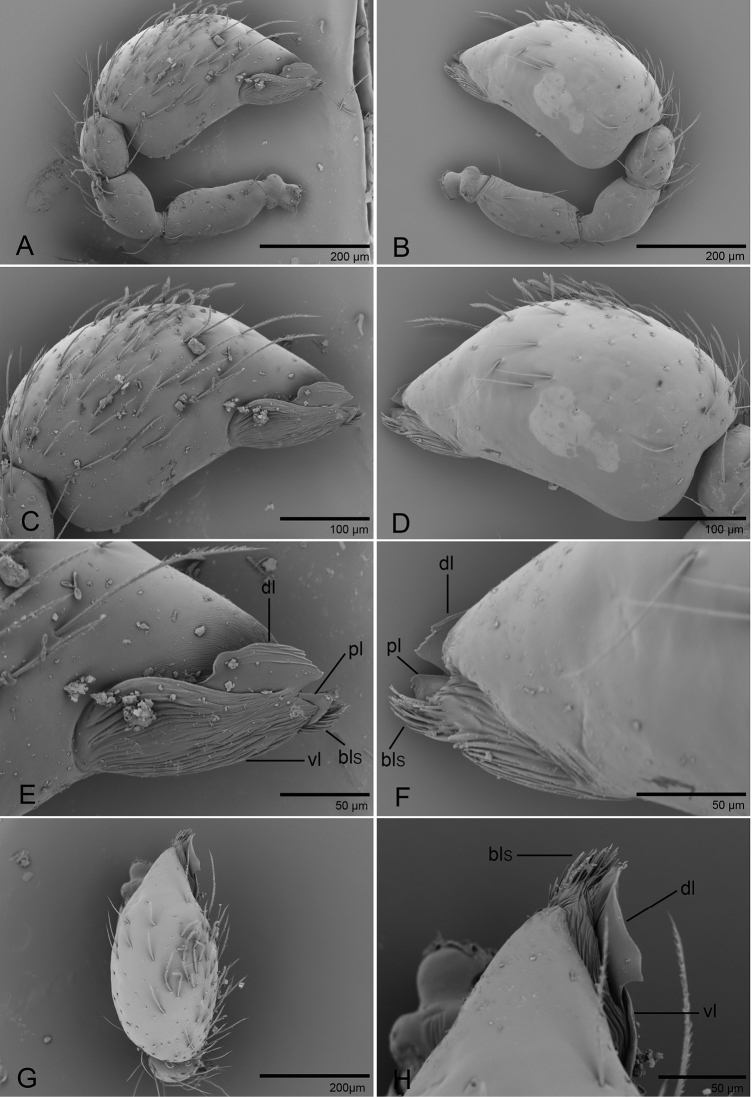
*Promolotra shankhaung* sp. nov., male left palp, SEM**A, B** prolateral and retrolateral views **C, D, G** cymbiobulbus, prolateral, retrolateral, and dorsal views **E, F, H** distal part of cymbiobulbus, prolateral, retrolateral, and dorsal views. Abbreviations: bls = brush-like structures; dl = dorsal lobe; pl = posterior lobe; vl = ventral lobe.

**Figure 3.**
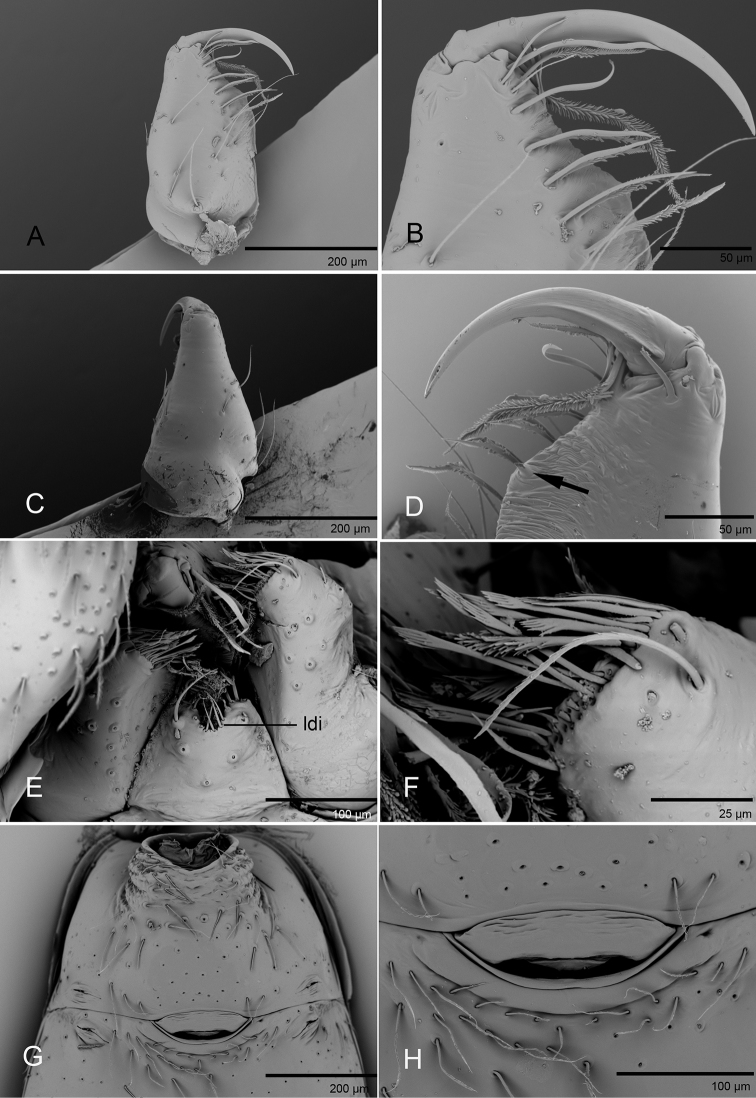
*Promolotra shankhaung* sp. nov. **A–F** male **G, H** female, SEM**A, C** left chelicera, anterior and lateral views **B, D** left chelicera, anterior and posterior magnified views (arrow shows the large denticle) **E** labium and endites, ventral view **F** endite, ventral view **G, H** copulatory organ, ventral view. Abbreviation: ldi = labium deep incision.

**Figure 4.**
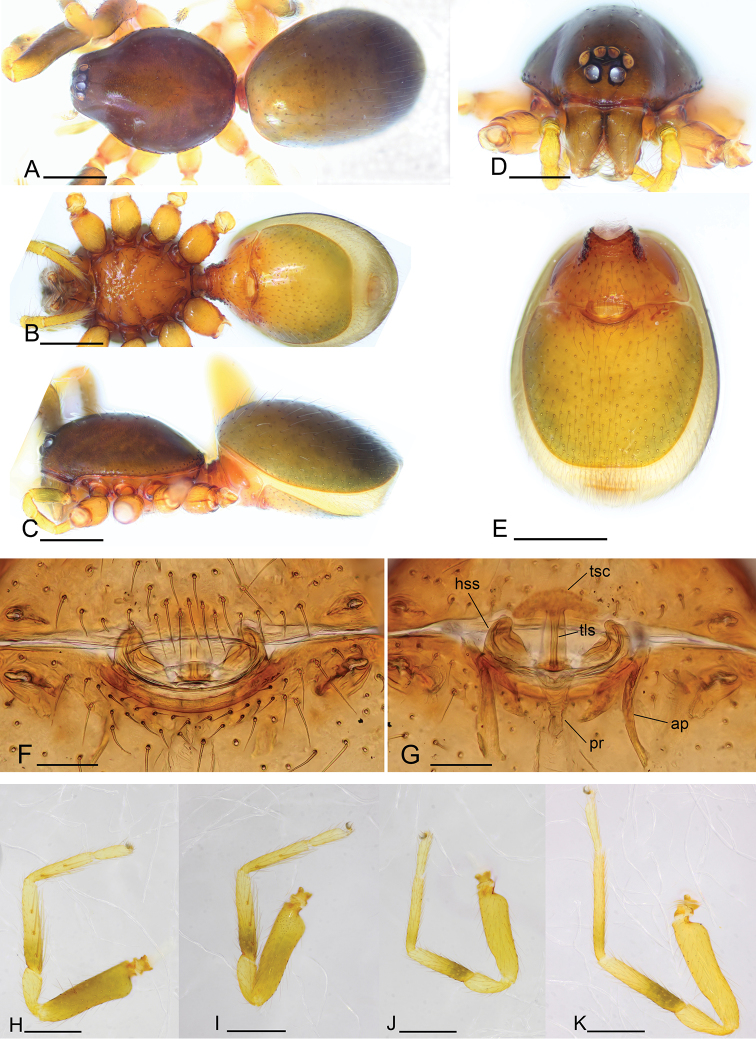
*Promolotra shankhaung* sp. nov. **A–G** female **H–K** male **A–C** habitus, dorsal, ventral, and lateral views **D** prosoma, anterior view **E** abdomen, ventral view **F, G** copulatory organ, ventral and dorsal views **H–K** left legs I–IV, prolateral view. Abbreviations: ap = apodeme; hss = horseshoe-shaped sclerite; pr = posterior receptaculum; tls = tube-like structure; tsc = T-shaped sclerite. Scale bars: 0.4 mm (**A–C, E, H–K**); 0.2 mm (**D**); 0.1 mm (**F, G**).

**Figure 5.**
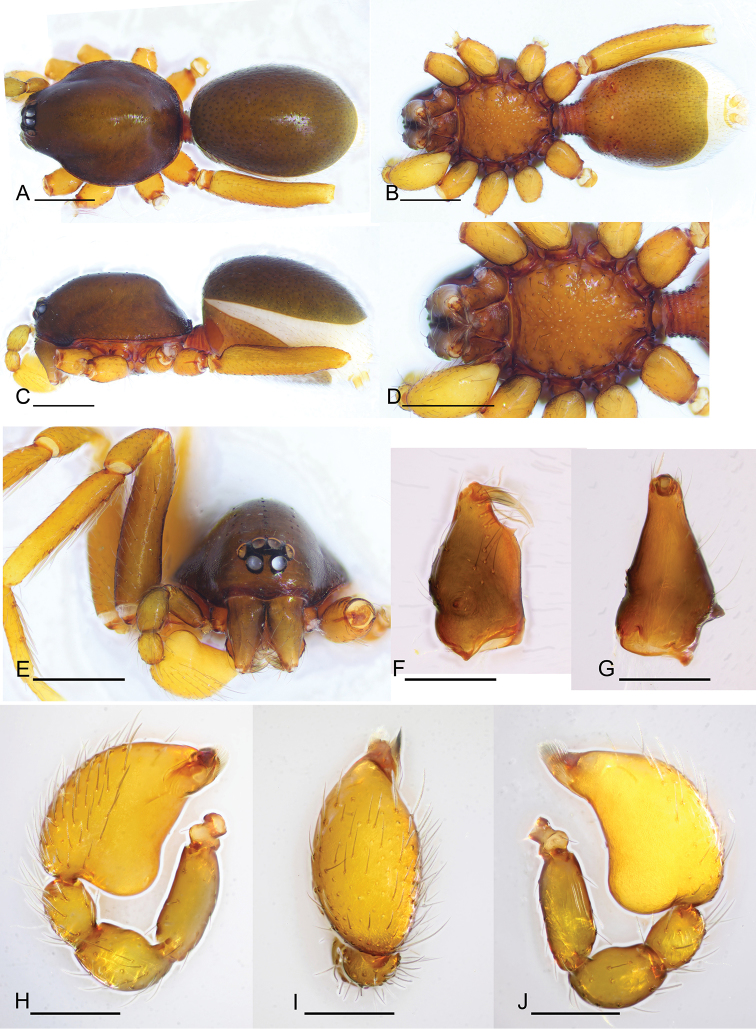
*Promolotra hponkanrazi* sp. nov., male **A–C** habitus, dorsal, ventral, and lateral views **D** prosoma, ventral view **E** prosoma, anterior view **F, G** left chelicera, anterior and lateral views **H–J** left palp, prolateral, dorsal, and retrolateral views. Scale bars: 0.4 mm (**A–E**) ; 0.2 mm (**F–J**).

**Figure 6.**
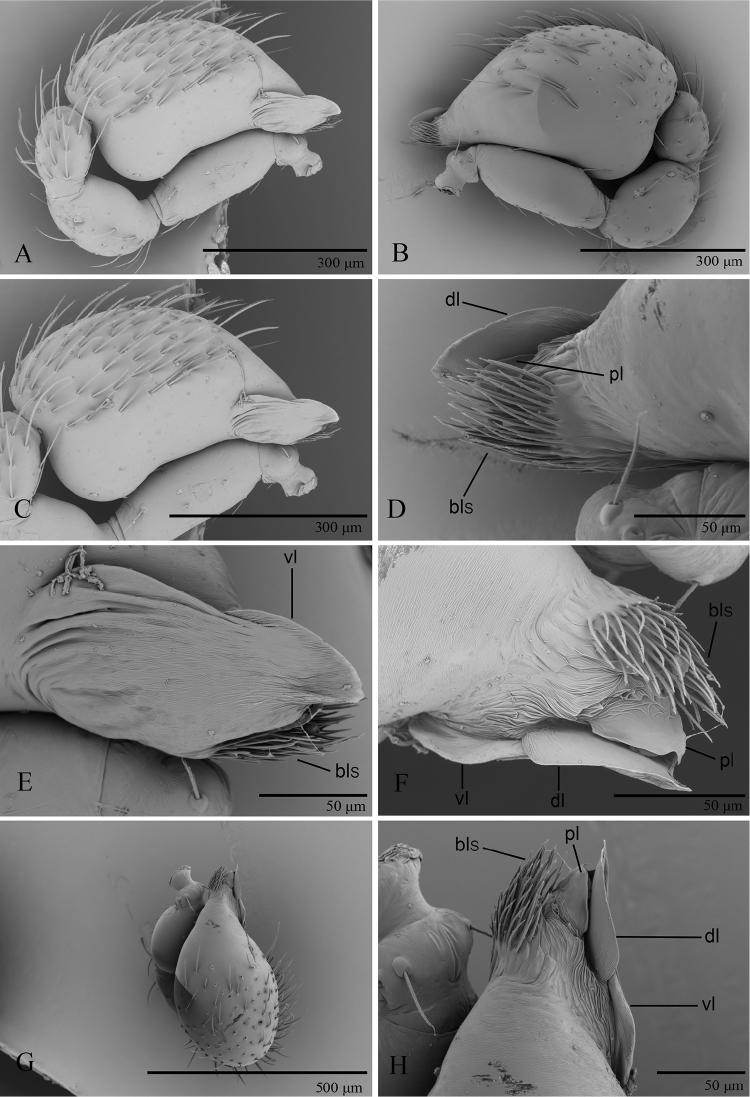
*Promolotra hponkanrazi* sp. nov., male left palp, SEM**A, B** prolateral and retrolateral views **C, G** cymbiobulbus, prolateral and dorsal views **D, E, H** distal part of cymbiobulbus, retrolateral, prolateral, and dorsal views **F** distal part of cymbiobulbus, apical view. Abbreviations: bls = brush-like structures; dl = dorsal lobe; pl = posterior lobe; vl = ventral lobe.

**Figure 7.**
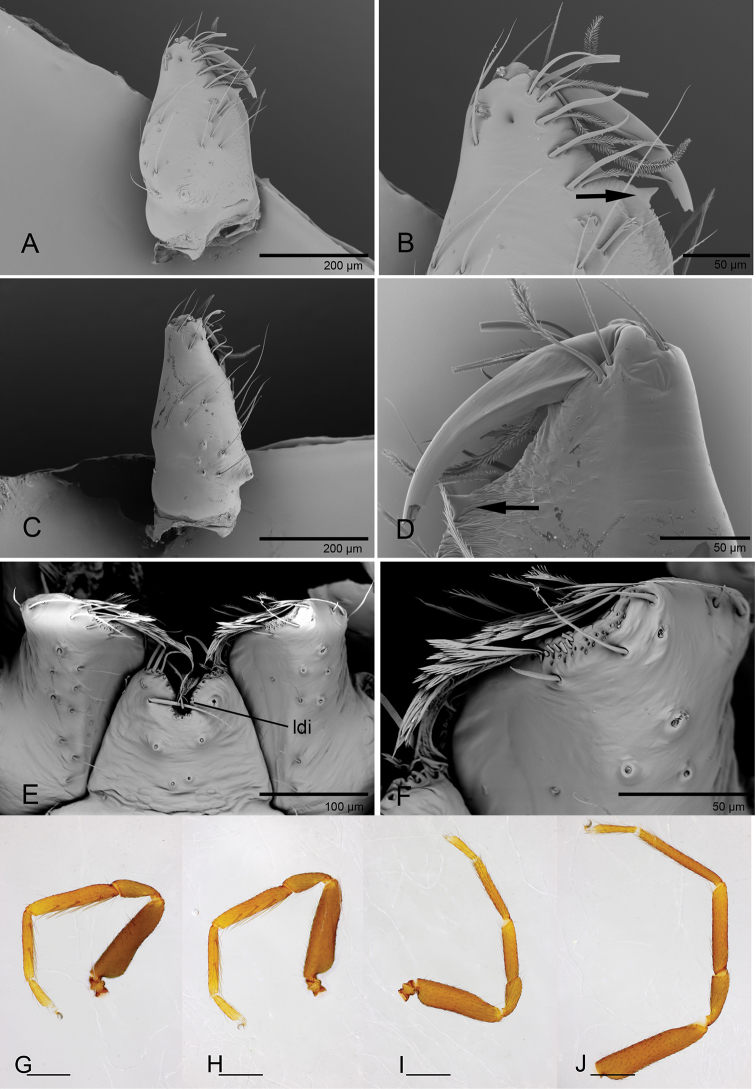
*Promolotra hponkanrazi* sp. nov., male **A–F**SEM**A, C** left chelicera, anterior and lateral views **B, D** left chelicera, anterior and posterior magnified views (arrows show the large denticle) **E** labium and endites, ventral view **F** endite, ventral view **G–J** right legs I–IV, prolateral view. Abbreviation: ldi = labium deep incision. Scale bars: 0.4 mm (**G–J**).

**Male.** Body yellow-brown, legs yellow. Carapace (Figs 1A, 5A): broadly oval in dorsal view, without any pattern; pars cephalica slightly elevated in lateral view, with rounded posterolateral corners, posterolateral edge without pits, posterior margin not bulging below posterior rim, anterolateral corners without extensions or projections, posterolateral surface without spikes, surface of pars cephalica smooth, thorax without depressions, fovea absent, without radiating rows of pits; lateral margin straight, rebordered, with small blunt denticles; marginal setae present. Eyes (Figs 1A, E, 5A, E): 6, well-developed, arranged in a compact group; ALE largest, PME, PLE subequal; ALE separated by nearly more than their radius, ALE–PLE separated by less than ALE radius, PME touching each other; posterior row recurved from above, procurved from front. Clypeus (Figs 1E, 5E): margin unmodified, sinuous in front view, vertical in lateral view; ALE separated from edge of carapace by 2 times their diameter. Chilum absent. Mouthparts (Figs 3A–F, 7A–F): chelicerae straight, anterior face strongly swollen, with cone-shaped protuberance in lateral view (Figs 1G, 3C, 5G, 7C); with large tooth on promargin; labium rectangular, anterior margin deeply incised (ldi), same as sternum in sclerotization, not fused to sternum; endites with distal excavation, posteromedian part unmodified, same as sternum in sclerotization. Sternum (Figs 1B, 5D): uniformly orange-brown, not fused to carapace, median concavity absent; longer than wide, with radial furrows between coxae, surface smooth, covered with small, round pits, anterior margin unmodified, posterior margin not extending posteriorly of coxae IV, anterior corner unmodified, distance between coxae approximately equal, lateral margins unmodified, without posterior hump; setae sparse, dark, needlelike, evenly scattered, without hair tufts. Abdomen (Figs 1B, C, 5A–C): ovoid, rounded posteriorly; booklung covers large, brown, without setae, anterolateral edge unmodified; pedicel tube medium-sized, ribbed, scutum not extending far beyond dorsum of pedicel, lacking plumose hairs. Sperm pore small, oval, rebordered, situated between anterior and posterior spiracles; anterior and posterior spiracles not connected by grooves. Dorsal scutum strongly sclerotized, orange-brown, without pattern, covering full length of abdomen, no soft tissue visible from above, separate from epigastric scutum. Epigastric scutum strongly sclerotized, surrounding pedicel. Postgastric scutum strongly sclerotized, covering nearly full length of abdomen, fused to epigastric scutum, anterior margin unmodified, with posteriorly directed lateral apodemes. Spinneret scutum present as incomplete ring, with fringe of setae. Colulus represented only by setae. Legs (Figs 1D, 4H–K, 7G–J): yellowish brown, with brown pattern on basal part of tibiae in *P. shankhaung* sp. nov.; patella plus tibia I shorter than carapace. Trichobothria - tibia: each with 3; metatarsus: each with 1. Leg spines: tibiae I–II with 4 pairs of ventral spines; metatarsi I–II with 2 pairs of ventral spines, legs III and IV without spines. Palp (Figs 1H–J, 2, 5H–J, 6): normal size, weakly sclerotized, right and left palps symmetrical, uniformly pale orange. Trochanter unmodified; femur normal size, 3 or more times as long as trochanter; patella shorter than femur, without prolateral row of ridges. Cymbium completely fused with bulb, not extending beyond distal tip of bulb. Embolar region (Figs 2E, F, H, 6D, E, F, H) consists of 3 broad lobes and brush-like structures.

**Female.** As in male except as noted. Abdomen (Fig. 4E): postgastric scutum rectangular, not fused to epigastric scutum. Copulatory organ: surface with conspicuous genital atrium (Fig. 3G, H). Dorsal view (Fig. 4G) with a T-shaped sclerite (tsc) anteriorly, followed posteriorly by a narrow posterior receptaculum (pr); lateral apodemes (ap) present.

#### Composition.

*P. hponkanrazi* sp. nov. (♂♀) and *P. shankhaung* sp. nov. (♂).

#### Distribution.

Myanmar (Kachin State).

### Promolotra shankhaung sp. nov.

Taxon classificationAnimaliaAraneaeOonopidae

AB65DC8D-F77A-5B47-B760-92A13A9A7121

http://zoobank.org/A21E734C-1501-4AB5-9906-B0CFB717874B

Figures 1 , 2 , 3 , 4

#### Type materials.

***Holotype*** ♂ (IZCAS Ar-25131), Myanmar, Kachin State, Putao, roadside between Upper Shankhaung Village to Wasadum, secondary forest, 27°27.383'N, 97°13.650'E, elevation ca 1396 m, 11.XII.2016, J. Wu, by hand. ***Paratypes***: 1♂, 1♀ (IZCAS Ar-25132-25133), same data as holotype; 1♂ (IZCAS Ar-25134), same data as holotype; 1♂, 1♀ (IZCAS Ar-25135-25136), Myanmar, Kachin State, Putao, roadside between Upper Shankhaung Village to Wasadum, 27°28.350'N, 97°12.850'E, elevation ca 1140 m, 11.XII.2016, J. Wu, by hand; 1♀ (IZCAS Ar-25137), Myanmar, Kachin State, Putao, Hponkanrazi Wildlife Sactuary, near Ziradum Village, 27°35.305'N, 97°04.893'E, elevation ca 1145 m, 13.V.2017, J. Wu and Z. Chen, by hand.

#### Etymology.

The specific name is a noun in apposition taken from the type locality.

#### Diagnosis.

The new species is similar to *P. hponkanrazi* sp. nov. (known from male only), but can be distinguished by the dark proximal part of the tibiae (Fig. 1D) (vs. uniformly colored in *P. hponkanrazi* sp. nov.), the relatively narrow ventral lobe (length/width = 2.4) of the embolar region (Fig. 2E) (vs. length/width of ventral lobe = 2.0 in *P. hponkanrazi* sp. nov.), and the shape of the cymbiobulbus (length/width = 1.7, basal part smooth) (Figs 1H, J, 2A) (vs. length/width of cymbiobulbus = 1.5 with basal part strongly swollen in *P. hponkanrazi* sp. nov.).

#### Description.

**Male** (holotype). Habitus as in Fig. 1A–C. Body length 1.95; carapace 0.96 long, 0.74 wide; abdomen 0.89 long, 0.72 wide.

Palp (Figs 1H–J, 2): femur 0.19 long, patella 0.13 long, tibia 0.12 long. Cymbiobulbus 0.38 long, 0.22 wide, length/maximal width = 1.7. Embolar region (Fig. 2E, F, H): including a flat dorsal lobe (dl), a small posterior one (pl), and a narrow (length/width = 2.4), leaf-like, wrinkled texture ventral one (vl); with brush-like structures (bls) in retrolateral view.

**Female.** Habitus as in Fig. 4A–C. Body length 2.23; carapace 1.01 long, 0.91 wide; abdomen 1.17 long, 0.85 wide.

Copulatory organ. Ventral view (Figs 3G, H, 4F): genital atrium relatively wide, broadly oval. Dorsal view (Fig. 4G): with a T-shaped sclerite (tsc) anteriorly, followed posteriorly by a narrow posterior receptaculum (pr); a very thin, long and tube-like structure (tls) can be seen inside the T-shaped sclerite; with a horseshoe-shaped sclerite (hss) medially; apodemes (ap) well-developed.

#### Distribution.

Known only from the type locality.

### Promolotra hponkanrazi sp. nov.

Taxon classificationAnimaliaAraneaeOonopidae

DBF67C7A-ECFB-5969-915E-F9AB953C7E85

http://zoobank.org/630E87B0-7B79-4531-93C3-85421EEEAA0F

Figures 5 , 6 , 7

#### Type materials.

***Holotype*** ♂ (IZCAS Ar-25138), Myanmar, Kachin State, Putao, Hponkanrazi Wildlife Sactuary, secondary forest, 27°36.867'N, 96°58.933'E, elevation ca 2491 m, 15.XII.2016, J. Wu, by hand.

#### Etymology.

The specific name is a noun in apposition taken from the type locality.

#### Diagnosis.

The new species is similar to *P. shankhaung* sp. nov. but can be distinguished by the uniformly colored tibiae (Figs 5E, 7G–J) (vs. darkened proximally in *P. shankhaung* sp. nov.), the relatively broad ventral lobe (length/width = 2.0) of embolar region (Fig. 6E) (vs. length/width of ventral lobe = 2.4 in *P. shankhaung* sp. nov.), and the shape of the cymbiobulbus (length/width = 1.5, basal part strongly swollen) (Figs 5H, J, 6A) (vs. length/width of cymbiobulbus = 1.7 and the basal part smooth in *P. shankhaung* sp. nov.).

#### Description.

**Male** (holotype). Habitus as in Fig. 5A–C. Body length 2.26; carapace 1.09 long, 0.85 wide; abdomen 1.13 long, 0.77 wide.

Palp (Figs 5H–J, 6): femur 0.26 long, patella 0.19 long, tibia 0.14 long. Cymbiobulbus 0.42 long, 0.28 wide, length/maximal width = 1.5. Embolar region (Fig. 6D, E, F, H): including a flat, dorsal lobe (dl), a very small posterior one (pl), and a very broad (length/width = 2.0), leaf-like, elongated, wrinkled texture ventral one (vl); with brush-like structures (bls) in retrolateral view.

**Female.** Unknown.

#### Distribution.

Known only from the type locality.

## Supplementary Material

XML Treatment for Promolotra

XML Treatment for Promolotra shankhaung

XML Treatment for Promolotra hponkanrazi
